# How do we measure the costs, benefits, and harms of sharing data from biomedical studies? A scoping review

**DOI:** 10.12688/openreseurope.16063.1

**Published:** 2023-09-18

**Authors:** Lauren Maxwell, Priya Shreedhar, Ankur Krishnan

**Affiliations:** 1 Heidelberger Institut für Global Health, Universitätsklinikum Heidelberg, Heidelberg, Baden-Württemberg, 69120, Germany

**Keywords:** Scoping review, data reuse, research translation, research impact, ethics review committee, biomedical research, data sharing, secondary research

## Abstract

Introduction

The benefits of sharing participant-level data from biomedical studies have been widely touted and may be taken for granted. As investments in data sharing and reuse efforts continue to grow, understanding the cost and positive and negative effects of data sharing for research participants, the general public, individual researchers, research and development, clinical practice, and public health is of growing importance. In this scoping review, we will identify and summarize existing evidence on the positive and negative impacts and costs of data sharing and how they are measured.

Methods and analysis

Eligible studies will report on qualitative or quantitative approaches for measuring the cost of data sharing or its impact on participant privacy, individual or public health, researcher’s careers, clinical or public health practice, or research or development. The systematic search strategy uses MeSH and text terms and is tailored for Ovid Medline, Cumulative Index to Nursing and Allied Health Literature (CINAHL), and Web of Science. We will apply the Arskey and O’Malley scoping review methodology. We selected a scoping rather than a systematic review approach to address multiple related questions and provide guidance related to an emerging field. Two reviewers will conduct the title-abstract and full-text screening and data charting independently. Discrepancies will be resolved through consensus and results will be summarized in a narrative form.

Conclusion

Research participants, investigators, regulatory groups, ethics review committees, data protection officers, and funders cannot make informed decisions or policies about data and sample reuse without appropriate means of measuring the effects, positive or negative, and cost of data and sample sharing.

## Introduction

Biomedical research funders and journals increasingly require that investigators share data and samples generated through research ^1, 2^. Sharing data from biomedical research is expected to prevent unnecessary research, improve research transparency, reduce drug development and approval timelines, inform the development and evaluation of randomized controlled trials (RCTs), and improve clinical and public health inference ^2– 4^. Despite the focus on the importance of data sharing and requirements for data sharing by funders, institutions, or journals, the costs and benefits of data sharing for researchers, research participants, and their source communities, the Open Science community, and the public at large are not well understood. Research participants, researchers, ethics review committee (ERC) members, data protection officers (DPOs), or other institutional stakeholders may have concerns about the adverse effects of data or sample sharing or its cost ^5– 9^. The benefits of data sharing are described qualitatively through case studies that may have been selected to prove that data sharing is beneficial ^10^.

While the benefits of sharing participant-level data are frequently highlighted by funders, journals, and others, measuring and addressing the risk and harms associated with data and sample sharing is equally important. Costs and harms related to data and sample sharing include: the cost to research teams associated with generating the metadata, cleaning and potentially harmonizing the dataset, and ensuring ethical data sharing ( *e.g.* broad consent, waiver of broad consent), which may result in reduced time for their own publications or funding applications; misinterpretation of data by teams that are using but did not collect the data, potentially leading to misleading findings and inappropriate public health recommendations; helicopter research when researchers that are reusing the data fail to appropriately credit the team that collected the data; benefits from data reuse that do not accrue to the study source population; exploitation of the data for commercial purposes without benefit sharing with the research team or source population; unethical or otherwise inappropriate handling of incidental findings; identification of research participants; and broad use of the data without receipt of broad consent for future use or a waiver of consent ^7, 9, 11– 13^. Quantifying the actual risk of these harms may help researchers and ERCs be more open to sharing participant-level data.

Data and sample sharing may be more likely to benefit researchers in high-income countries and disadvantage researchers in low-and-middle-income countries, leading to an inequitable distribution of benefits and harms related to data reuse ^14^. In contrast, not sharing data or samples can lead to missed opportunities for improved development of medications and vaccines or informed clinical and public health decision-making. Understanding how benefits and harms are measured can help strengthen their measurement to help ensure equitable reuse.

### Study rationale and objectives

This scoping review will identify and describe qualitative and quantitative approaches to measuring the impact of data sharing on researchers’ careers, clinical or public health practice, and science. We will examine the types of studies and the results of those studies related to the impacts and costs of data sharing. Data sharing will be defined broadly as individual investigators sharing data on a case-by-case basis or individuals or institutions sharing data on a cross-project level through individual participant data meta-analyses (IPD-MAs), data verses, data lakes, platforms, repositories, and registries. The information collected and summarized through this scoping review will be used to develop a research agenda for measuring the harms, benefits, and costs of data sharing.

Within public health research, data sharing varies by data type. While sharing high dimensional genetic data is expected, enforced, and facilitated by widely adopted data and metadata standards, sharing other data types, like clinical-epidemiological data, is less common and, in some ways, more complex. In this review, we focus on the benefits of sharing participant-level clinical-epidemiological data from longitudinal studies, whether observational or intervention studies.

Biomedical research conducted with data from electronic health records (EHRs) is substantively different than original data collection because data do not need to be transformed unless researchers put together multiple EHRs, so data are reused with minimal cost to the investigator. As such, we will not include studies that reuse EHR data from one system, where data reuse costs are limited.

## Methods

Scoping reviews provide a broad overview of existing literature and help identify priority areas for future research investment ^15^. The scoping review questions listed below are based on a literature review, consultation with subject matter experts, and the review team’s prior and ongoing work on data sharing. In keeping with the iterative nature of a scoping review, the questions will be updated based on review findings as needed. This scoping review protocol was developed in keeping with the PRISMA-P guidelines ^16^. This scoping review will report findings per the PRISMA Extension for Scoping Reviews guidance (PRISMA-ScR) ^17^. The scoping review will follow Levac *et al.*’s recommendations for the conduct of scoping reviews ^18^:

Stage 1. clarify and link the purpose and research question (balancing)
Stage 2. apply an iterative approach to developing and evaluating the comprehensiveness of the scoping process
Stage 3. define the method of study selection
Stage 4. clarify the approach to data extraction
Stage 5. qualitative thematic analysis, summarizing results, and describing policy, practice, and research implications
Stage 6. stakeholder consultation to summarize results and facilitate research translation

### Stage 1: Identifying the research question

The initial review questions were developed through consultation with the research team and feedback from researchers and stakeholders, including funding groups, who work to facilitate data reuse. The scoping review will cover both the measurement of data sharing and evidence for its short- and long-term costs, benefits, and harms. The scoping review questions include the following:

***Measurement***

1. How do reports or publications related to the benefits, harms, or costs of data sharing measure data sharing?

i. Case studies, empirical measures

2. Is there a lot of difference between question 1 and 2?

3. How is the cost of data sharing determined?

***Empirical evidence***

1. What evidence do we have to support data-sharing-related fears?

i. Lost opportunities for funding or publication
ii. Identification and stigmatization of individual research participants
iii. Misinterpretation of data

2. What evidence do we have to support data-sharing-related benefits?

i. New insights
ii. Increased opportunities for funding or publication for individual researchers or research groups that share or reuse data ( *e.g.*, publication rate, publication impact factor)
iii. New collaborations or areas of inquiry for individuals or groups that share or reuse data ( *e.g.*, citation network)
iv. Improved study design
v. Translation or more rapid translation of research findings into practice
vi. Prevention of unnecessary research

3. What evidence do we have about the cost or savings associated with sharing or not sharing data for investigators, institutions, and funders?

### Stage 2: Identifying relevant studies

We will examine grey literature ( *e.g*., case reports from funders, research, or public health institutions) and peer-reviewed studies. We will systematically search the following electronic databases: Ovid (Medline), Cumulative Index to Nursing and Allied Health Literature (CINAHL), and Web of Science. We developed a text-based search strategy tailored to each database, with MeSH terms used for the Ovid (Medline) and CINAHL searches. We will search OpenGrey, Google, and the websites of national or international bodies whose work focuses on data sharing ( *e.g*., Wellcome Trust, RAND corporation) to identify related grey literature. Search results will be imported to EndNote X9, and duplicates will be removed. We will apply snowball sampling wherein we search the reference lists of included studies or related systematic reviews and scoping reviews for additional eligible studies. The Ovid (Medline) search strategy is presented in Table 1.

**Table 1.** Ovid (Medline) search strategy.

1.	(data sharing or data reuse or Datasets as Topic).mp. and (exp *Information Dissemination/ or exp *“Information Storage and Retrieval”/ or exp *Databases, Genetic/ or exp Registries/)
2.	((shar* or public or open or participant*level or patient*level or cross-study or reuse or reuse or use* or federat* or swarm or warehouse or lake) adj3 (data or research or sequence* or information or metadata) or database or datalake or data lake or data mesh or datamesh or repository or registry or platform).ti,ab.
3.	1 and 2
4.	(impact* or improv* or effect* or benefit* or advance* or translation or chang* or innovate* or novel or new or emerg* or build* or gain* or progress* or promot* or strength* or cultivat* or encourage* or improv* or breakthrough* or discover* or accelerat* or intervent* or headway* or success* or facilitat* or proceed or trust).ti,ab.
5.	(harm* or prevent* or stymie* or obviate or end or loss or impair* or misuse* or hurt or abuse* or injure* or block* or arrest*). ti,ab.
6.	(economic* or cost* of financ* or reimburs* or (health adj1 polic*) or (resource adj1 allocat*) or expenditure*).ti,ab.
7.	cost analysis.mp. or exp “Costs and Cost Analysis”/
8.	4 or 5 or 6 or 7
9	exp Biomedical Research/ or exp Medical Records/
10.	(health or patient or biomedical or medical or trial* or RCT* or intervention* or clinical or gene* or metabolomic* or genomic* or proteomic* or immunomic* or transcriptomic* or imag*).ti,ab.
11.	9 or 10
12	3 and 8 and 11
13.	limit 12 to humans
14.	(addresses or biography or case reports or comment* or directory or editorial or festschrift or interview or lectures or legal cases or legislation or letter* or editorial or news or newspaper article or patient education handout or popular works or congresses or consensus development conference or practice guideline).pt.
15.	13 not 14

### Stage 3: Study selection and eligibility criteria

Two reviewers will conduct the title and abstract screening and full-text review independently. We will update the search strategy if needed to explore emerging questions identified during the title/abstract screening or full-text review per the iterative nature of a scoping review. Title-abstract screening, full-text screening, and data extraction will be managed in Covidence (RRID:SCR_016484) ^19^. Inter-reviewer disagreements in the title-abstract or full-text screening will be resolved by consensus.

***Inclusion criteria***

Included studies will be research or reports that describe approaches to measuring the cost, harms, or benefits of sharing or otherwise reusing, as through federated learning, biomedical research data. We will not limit studies by geography or language of publication.

***Exclusion criteria***

Studies or reports that focus solely on sample rather than data sharing or reusing EHR data from one system, where there is minimal cost associated with data reuse because data do not need to be harmonized, will be excluded from the review. Editorials or commentaries will be excluded.

### Stage 4: Charting the data

We will develop and pilot a data extraction form with three included articles to facilitate the descriptive synthesis of scoping review findings. Table 2 presents preliminary data extraction fields that will be updated during the scoping review. We will not assess the quality of included studies.

**Table 2.** Data charting fields.

Study information	• Author • Publication year • Study design (e.g., case study, observational, intervention. qualitative, quantitative, mixed methods) • Main objective or research question • Funder
Population (for primary research studies)	• Location • Year of study, year of publication • Participant enrollment criteria • Source population • Research question • Selection process
Measurement of data sharing	• What data are shared • Quantity of data (N, size) • Marker of dataset value • FAIRness of dataset • Marker of data quality • When data are shared • In real-time or close to real-time • After finishing data collection • After the publication of related papers • With whom data are shared • How data are shared • Sent between investigators • Uploaded to a data lake or data verse (minimal effort) • Uploaded to registry or platform that requires harmonization (significant effort) • Trusted research environment (can be harmonized or not) • Measurement method • Subjective measures ○ Personal communication ○ A longitudinal or cross-sectional survey ○ Data sharing statements • Objective measures ○ Datasets uploaded ○ Datasets downloaded ○ Citations of dataset
Measurement of costs of data sharing	• Short and long-term cost or cost savings • Cost of harm to individuals, researchers, universities, health authorities or cost saving related to benefits to individuals, research groups, universities, health authorities
Measurement of benefits of data sharing	• What is the impact of data sharing on: • Researchers’ careers and institutions • Funding (new funders in new domains or geographies, number of grants, the amount received) • Publication rate • Impact factor of journals manuscripts published in • Research breadth and depth • Prevent spurious research & associated savings in funding/research community burnout • Lead to new fields of inquiry • Individual research participants or patients or their communities • Improving clinical care or diagnosis of rare diseases • Improved access to diagnostics, prophylactics, treatments
Measurement of harms of data sharing	• What are the adverse effects of data sharing on: • Researchers’ careers, institutions • Funding (number of grants, amount received) • Publication rate • Impact factor of journals manuscripts published in • Research breadth and depth • Spurious research & associated costs to funding/research community burnout • Cost to researchers of implementing FAIR, governance development, repository deposition • Individual research participants or patients or their communities: • Reidentification of individuals and groups • Harms associated with reidentification
Sources of bias	• Selection of study population (e.g., how are case studies selected) • Self-report versus verifiable metrics • Reporting method
Stakeholder concerns	• ERCs, funders, DPOs, patient advocacy groups, MoHs, and cross-national health authorities (e.g., WHO, US CDC, E-CDC)
Evidence gaps	• Summarize areas for future research

E-CDC, European Centre for Disease Prevention and Control; ERCs, ethics review committees; DPOs, data protection officers; FAIR, findable, accessible, interoperable, and reusable; MoHs, ministries of health; US CDC, United States Centers for Disease Control and Prevention; WHO, World Health Organization.

### Stage 5: Collating, summarising, and reporting results

We will report on the screening and inclusion process using a PRISMA diagram. The data charting work will be summarized in tables and narrative form. The results summary will present both quantitative and qualitative aspects of included studies.

### Stage 6: Consultation, patient & public involvement

We will present the summary of findings from this review to a panel of stakeholders, including research funders, policymakers, and journals, to develop priority areas for improving data-sharing metrics. We will share research findings with the general public through a webinar and the websites of partner institutions.

## Study status

We present the scoping review timeline in Table 3.

**Table 3.** Scoping review status at time of protocol registration and anticipated timeline.

Preliminary search	Completed
Pilot of search strategy	Completed
Implementation of search	June 2023
Title-abstract screening	June 2023
Full text screening	July 2023
Development and piloting of data charting tool	July-August 2023
Charting the data	August-October 2023
Publication of results	January 2024

## Conclusion

In this scoping review, we will identify and characterize measures of data sharing and its related costs, benefits, and harms to research participants and their source communities, researchers and their institutions, health authorities, health research funders, the Open Science community, and the general public. Costs of data sharing could include lost opportunities for publication, identification of research participants, and misinterpretation of the data. The benefits of data sharing may consist of ensuring the reliability and reproducibility of studies, identifying gaps in the research landscape, and improving the evidence base used to make regulatory, funding, and clinical decisions.

If we cannot measure and describe the costs, benefits, and harms of data reuse, we as researchers cannot inform patients and participants about the risks and benefits of data reuse, funders cannot identify and support successful data reuse efforts, and regulatory authorities, including ERCs and DPOs, cannot identify and address harms. This scoping review will summarize the existing state of research as a much-needed first step towards developing actionable, verifiable metrics for determining data sharing costs, benefits, and harms to maximize the return and minimize the harms of data sharing across stakeholder groups in the data reuse and research translation landscape.

## Ethics and dissemination

This scoping review summarizes findings from existing publications in peer-reviewed journals and does not require ethics review. Results will be disseminated through an open-access publication.

## Reporting guidelines

This scoping review protocol was developed in keeping with the PRISMA-P guidelines ^16^. This scoping review will report findings per the PRISMA-ScR guidelines ^17^.

## Data Availability

No data are associated with this article. Open Science Foundation: PRISMA-P checklist for “How do we measure the costs, benefits, and harms of sharing data from biomedical studies? A scoping review.” for https://osf.io/3dngr. **LM:** Conceptualization, Funding acquisition, Supervision, Methodology, Writing- Original draft preparation. **PS**: Writing- Review and Editing. **AK:** Methodology, Writing- Review and Editing.
