# How do we measure the costs, benefits, and harms of sharing data from biomedical studies? A protocol for a scoping review

**DOI:** 10.12688/openreseurope.16063.2

**Published:** 2025-01-14

**Authors:** Lauren Maxwell, Priya Shreedhar, Ankur Krishnan

**Affiliations:** 1 Heidelberger Institut für Global Health, Universitätsklinikum Heidelberg, Heidelberg, Baden-Württemberg, 69120, Germany

**Keywords:** Scoping review, data reuse, research translation, research impact, ethics review committee, biomedical research, data sharing, secondary research

## Abstract

**Introduction:**

The benefits of sharing participant-level data, including clinical or epidemiological data, genomic data, high-dimensional imaging data, or human-derived samples, from biomedical studies have been widely touted and may be taken for granted. As investments in data sharing and reuse efforts continue to grow, understanding the cost and positive and negative effects of data sharing for research participants, the general public, individual researchers, research and development, clinical practice, and public health is of growing importance. In this scoping review, we will identify and summarize existing evidence on the positive and negative impacts and costs of data sharing and how they are measured.

**Methods and analysis:**

Eligible studies will report on qualitative or quantitative approaches for measuring the cost of data sharing or its impact on participant privacy, individual or public health, researcher’s careers, clinical or public health practice, or research or development. The systematic search strategy uses MeSH and text terms and is tailored for Ovid Medline, Cumulative Index to Nursing and Allied Health Literature, and Web of Science. We will apply the Arskey and O’Malley scoping review methodology. We selected a scoping rather than a systematic review approach to address multiple related questions and provide guidance related to an emerging field. Two reviewers will conduct the title-abstract and full-text screening and data charting independently. Discrepancies will be resolved through consensus and results will be summarized in a narrative form.

**Conclusion:**

Research participants, investigators, regulatory groups, ethics review committees, data protection officers, and funders cannot make informed decisions or policies about data reuse without appropriate means of measuring the effects, positive or negative, and cost of data sharing.

## Introduction

Biomedical research funders and journals increasingly require that investigators share data and samples generated through research ^1, 2^. Sharing data, including clinical or epidemiological (clin-epi) data, genetic data, high-dimensional imaging data, or human-derived samples and related metadata, from biomedical research is expected to prevent unnecessary research, improve research transparency, reduce drug development and approval timelines, inform the development and evaluation of randomized controlled trials (RCTs), and improve clinical and public health inference ^2– 4^. Despite the focus on the importance of data sharing and requirements for data sharing by funders, institutions, or journals, the costs and benefits of data sharing for researchers, research participants, and their source communities, the Open Science community, and the public at large are not well understood. Research participants, researchers, ethics review committee (ERC) members, data protection officers (DPOs), or other institutional stakeholders may have concerns about the adverse effects of data or sample sharing or its cost ^5– 9^. The benefits of data sharing are described qualitatively through case studies that may have been selected to prove that data sharing is beneficial ^10^.

While the benefits of sharing participant-level data are frequently highlighted by funders, journals, and others, measuring and addressing the risk and harms associated with data and sample sharing is equally important. Costs and harms related to data and sample sharing include: the cost to research teams associated with generating the metadata, cleaning and potentially harmonizing the dataset, and ensuring ethical data sharing ( *e.g.* broad consent, waiver of broad consent), which may result in reduced time for their own publications or funding applications; misinterpretation of data by teams that are using but did not collect the data, potentially leading to misleading findings and inappropriate public health recommendations; helicopter research when researchers that are reusing the data fail to appropriately credit the team that collected the data; benefits from data reuse that do not accrue to the study source population; exploitation of the data for commercial purposes without benefit sharing with the research team or source population; unethical or otherwise inappropriate handling of incidental findings; identification of research participants; and broad use of the data without receipt of broad consent for future use or a waiver of consent ^7, 9, 11– 13^. Quantifying the actual risk of these harms may help researchers and ERCs be more open to sharing participant-level data.

Data and sample sharing may be more likely to benefit researchers in high-income countries and disadvantage researchers in low-and-middle-income countries, leading to an inequitable distribution of benefits and harms related to data reuse ^14^. In contrast, not sharing data or samples can lead to missed opportunities for improved development of medications and vaccines or informed clinical and public health decision-making. Understanding how benefits and harms are measured can help strengthen their measurement to help ensure equitable reuse.

### Study rationale and objectives

This scoping review will identify and describe qualitative and quantitative approaches to (1) measuring the positive impacts of biomedical data or sample sharing or other forms of data or sample reuse (e.g. federated data or sample reuse) on researchers’ careers, clinical or public health practice, and science; (2) the harms of data or sample sharing, including harms to investigators, research participants or patients whose routinely collected data are reused for research, or public health practice as through misuse of data for misleading inference and (3) the costs associated with data and sample reuse. We will examine the types of studies and the results of those studies related to the impacts (e.g., positive, negative, no impact) and costs of data sharing. Biomedical data sharing will be defined broadly as individual investigators sharing data on a case-by-case basis or individuals or institutions sharing data on a cross-project level through individual participant data meta-analyses (IPD-MAs), data verses, data lakes, platforms, repositories, and registries. The information collected and summarized through this scoping review will be used to develop a research agenda for measuring the harms, benefits, and costs of biomedical data and sample sharing.

Within public health research, data sharing varies by data type. While sharing high dimensional genetic data is expected, enforced, and facilitated by widely adopted data and metadata standards, sharing other data types, like clinical-epidemiological data, is less common and, in some ways, more complex. As such, we might expect to see differences in the cost of sharing clin-epi rather than human or pathogen OMICs data.

## Methods

Scoping reviews provide a broad overview of existing literature and help identify priority areas for future research investment ^15^. The scoping review questions listed below are based on a literature review, consultation with subject matter experts, and the review team’s prior and ongoing work on data sharing. In keeping with the iterative nature of a scoping review, the questions will be updated based on review findings as needed. This scoping review protocol was developed in keeping with the PRISMA-P guidelines ^16^. This scoping review will report findings per the PRISMA Extension for Scoping Reviews guidance (PRISMA-ScR) ^17^. The scoping review will follow Levac *et al.*’s recommendations for the conduct of scoping reviews ^18^:

Stage 1. clarify and link the purpose and research question (balancing)
Stage 2. apply an iterative approach to developing and evaluating the comprehensiveness of the scoping process
Stage 3. define the method of study selection
Stage 4. clarify the approach to data extraction
Stage 5. qualitative thematic analysis, summarizing results, and describing policy, practice, and research implications
Stage 6. stakeholder consultation to summarize results and facilitate research translation

### Stage 1: Identifying the research question

The initial review questions were developed through consultation with the research team and feedback from researchers and stakeholders who participated in the Coalition for Equitable Research in Low Income Settings (CERCLE) in 2019, and with the 2019 Global Research Collaboration for Infectious Disease Preparedness (GloPID-R) Data Sharing Working Group membership, who work to facilitate biomedical data reuse in the context of infectious disease and beyond. Following these stakeholder consultations, the review scope was unchanged. The scoping review will cover both the measurement of data sharing and evidence for its short- and long-term costs, benefits, and harms. The scoping review questions include the following:

***1. Data Sharing-Related Benefits***

**Metrics and Evidence**

- **1.1. What types of metrics or methods are used to measure the benefits of biomedical data or sample sharing?**
  Examples:
  - Case studies, empirical measures, modelled estimates.
- **1.2. What are the sources of data used to measure these benefits?**
  Examples:
  - Researcher or funder self-report, bibliometrics, altmetrics, dataset persistent identifiers, scientific knowledge graphs (e.g., OpenAIRE, Web of Science).

**Empirical Evidence**

- **1.3. What evidence supports the benefits of biomedical data or sample sharing?**
  Examples:
  - New scientific or clinical insights.
  - New opportunities for research and development (e.g., patents).
  - Increased funding or publication opportunities.
  - New collaborations or expanded research networks.
  - Improved study design and translation of findings into practice.
  - Prevention of unnecessary research.

***2. Data Sharing-Related Harms***

**Metrics and Evidence**

- **2.1. What types of metrics or methods are used to assess the harms of biomedical data or sample sharing?**
  Examples:
  - Empirical measures, case studies, modelled estimates.

**Empirical Evidence**

- **2.2. What evidence supports concerns or fears about biomedical data or sample sharing?**
  Examples:
  - Lost opportunities for funding or publication.
  - Identification and stigmatization of participants.
  - Misinterpretation or misuse of data or samples.
  - Lack of benefit sharing with source communities.
  - Ethical concerns, especially for indigenous or marginalized populations.
  - Lack of credit or attribution for data or sample providers.

***3. Data Sharing-Related Costs and Savings***

**Metrics and Evidence**

- **3.1. What types of metrics or methods are used to assess the costs and savings of biomedical data or sample sharing?**
  Examples:
  - Modelled estimates, cost-benefit analyses.
- **3.2. What are the sources of data used to evaluate costs and savings?**
  Examples:
  - Institutional financial records, funding reports, researcher self-report.

**Empirical Evidence**

- **3.3. What evidence exists about the costs or savings associated with sharing or not sharing biomedical data or samples?**
  Examples:
  - Direct and indirect costs for investigators, institutions, and funders.
  - Cost-effectiveness or savings from avoided duplication of research.

### Stage 2: Identifying relevant studies

We will examine grey literature, including case reports from public funders of biomedical, research, or public health institutions and peer-reviewed studies. We will systematically search the following electronic databases: Ovid (Medline), Cumulative Index to Nursing and Allied Health Literature (CINAHL), and Web of Science (Core Collection). We developed a text- and MeSH-term-based search strategy tailored to each database, with MeSH terms used for the Ovid (Medline) and CINAHL searches. In keeping with the Peer Review of Electronic Search Strategies (PRESS) 2015 Guidelines ^19^, the search strategies were developed through: translating the research questions into a PICO format, using Boolean and proximity operators, applying subject headings (e.g., MeSH terms) and free-text terms, pilot testing to ensure appropriate syntax, and applying limits and filters. The search strategies were reviewed by an information scientist at Universitätsklinikum Heidelberg Library.

We will search OpenGrey, Google, and the websites of national or international bodies whose work focuses on biomedical data sharing to identify related grey literature reports. These groups will include the Wellcome Trust, RAND corporation, Digital Research Alliance of Canada, Canadian Institutes of Health Research (CIHR), US National Institutes of Health, the European Commission, Fiocruz, Clinical Research Data Sharing Alliance (CRSDA), Research Data Alliance (RDA), World Data Systems (WDS), the Global Alliance for Genomics and Health (GA4GH), the European Bioinformatics Institute (EMBL-EBI) and the Biobanking and BioMolecular Resources Research Infrastructure (BBMRI-ERIC, the World Health Organization (WHO), CERCLE, the Infectious Diseases Data Observatory (IDDO), the European and Developing Countries Clinical Trials Partnership (EDCTP), the Global Health Network (TGHN), and the European Clinical .Research Infrastructure Network (ECRIN), the Bill and Melinda Gates Foundation (BMBF), the Terry Fox Foundation, the Rare Cancer Research Foundation (RCRF), the Alzheimer’s Disease Data Initiative (ADDI), the National Organization for Rare Disorders (NORD), the Haystack Project, the Cancer Research Data Commons (CRDC), Accelerating Medicines Partnership (AMP), the Observational Health Data Sciences and Informatics (OHDSI) initiative, The Future of Research Communication and e-Scholarship (FORCE11), and Make Data Count.

We will not search the websites of related biomedical data registries to understand what metrics these registries implement. A 6 December 2024 search of the FAIRsharing.org registry of databases indicates more the 1K databases in the biomedical space, which only considered databases that are registered on FAIRsharing.org ^20^, a small fraction of existing biomedical databases. Data reuse metrics from repositories and platforms tend to focus on the number of datasets shared and reused or the number of publications related to data reuse. In this review, our focus is on the benefits, harms, and cost of data reuse rather than on the quantity. The review and extraction of data reuse metrics from biomedical databases is beyond the scope of this work. Because of the possibility of biased reporting, we will not explore the websites of pharmaceutical companies or for-profit groups for their reports on the costs, benefits, and harms of biomedical data sharing and reuse. If reports on the costs, benefits, and harms of biomedical data reuse have been funded by for-profit groups, this information will be reported in the study description (e.g. Table 1 of the scoping review publication).

Searches will not be limited by date or language of publication. Search results will be imported to EndNote X9, and duplicates will be removed. We will apply snowball sampling wherein we search the reference lists of included studies or related systematic reviews and scoping reviews for additional eligible studies. The Ovid (Medline), CINAHL and Web of Science (Core Collection) search strategies are presented in Table 1a– Table 1c.

**Table 1a.** Ovid (Medline) search strategy.

1	exp Biomedical Research/ or exp Medical Records/ or exp Electronic Health Record or exp Biological Specimen Banks/
2	((health or patient or participant or study or cohort or biobank* or biorepositor* or biospecimen* or biosampl* or medical or trial* or RCT* or intervention* or clinical or gen* or metabo* or proteo* or immuno* or transcripto* or imag* or electronic) adj3 (data or research or sequence* or information or metadata or record*)).ti,ab.
3	(bio* adj2 (medical or sample* or specimen* or repositor* or bank*) adj3 (data or research or sequence* or information or metadata or record*)).ti,ab.
4	omic/.ti,ab.
5	1 and (2 or 3 or 4)
6	exp "Fluids and Secretions"/ or exp Specimen Handling/
7	((bio* adj2 (sample* or specimen* or fluid*)) or (swab* adj2 (throat or skin or cervic* or urethr* or conjuctiv*)) or (fluid* adj2 (pleur* or cerebr* or synov* or pericard* or amniot*)) or sample* or specimen* or biospecimen* or biosampl* or fluid* or blood or plasma or serum or urine or saliva or sputum or stool or biopsy or tissue or lavage or pus or marrow or bile or ascit* or semen or humor).ti,ab.
8	6 and 7
9	5 or 8
10	exp information dissemination/ or exp information management/ or exp data management/ or exp information services/ or "information storage and retrieval"/ or exp data warehousing/ or exp databases as topic/ or exp Registries/
11	((data or research or sequence* or information or metadata) adj3 (shar* or public or open or cross-study or access or request or retriev* or reuse or re-use or use* or federat* or swarm or warehouse or lake or base or mesh or repositor* or registr* or platform or management)).ti,ab.
12	10 and 11
13	exp *"costs and cost analysis"/ or *cost-benefit analysis/ or *cost-effectiveness analysis/ or *Resource Allocation/
14	(impact* or improv* or effect* or benefit* or advance* or translation or chang* or innovate* or novel or new or emerg* or build* or gain* or progress* or promot* or strength* or cultivat* or encourage* or improv* or breakthrough* or discover* or accelerat* or intervent* or headway* or success* or facilitat* or proceed or trust).ti,ab.
15	(harm* or prevent* or stymie* or obviate or end or loss or impair* or misuse* or hurt or abuse* or injure* or block* or arrest*).ti,ab.
16	(economic* or cost* of financ* or reimburs* or (health adj1 polic*) or (resource adj1 allocat*) or expenditure*).ti,ab.
17	13 or 14 or 15 or 16
18	9 and 12 and 17
19	limit 18 to humans
20	(addresses or biography or case reports or comment* or directory or editorial or festschrift or interview or lectures or legal cases or legislation or letter* or editorial or news or newspaper article or patient education handout or popular works or congresses or consensus development conference or practice guideline).pt.
21	19 not 20

**Table 1b.** CINAHL search strategy.

S1	TI ( ((health or patient or participant or study or cohort or biobank* or biorepositor* or biospecimen* or biosampl* or medical or trial* or RCT* or intervention* or clinical or gen* or metabo* or proteo* or immuno* or transcripto* or imag* or electronic) N3 (data or research or sequence* or information or metadata or record*)) ) AND AB ( ((health or patient or participant or study or cohort or biobank* or biorepositor* or biospecimen* or biosampl* or medical or trial* or RCT* or intervention* or clinical or gen* or metabo* or proteo* or immuno* or transcripto* or imag* or electronic) N3 (data or research or sequence* or information or metadata or record*)) )
S2	TI ( ((bio* N2 (medical or sample* or specimen* or repositor* or bank*) N3 (data or research or sequence* or information or metadata or record*)) ) AND AB ( ((bio* N2 (medical or sample* or specimen* or repositor* or bank*) N3 (data or research or sequence* or information or metadata or record*)) )
S3	TI *omic AND AB *omic
S4	TI ( ((bio* N2 (sample* or specimen* or fluid*)) or (swab* N2 (throat or skin or cervic* or urethr* or conjuctiv*)) or (fluid* N2 (pleur* or cerebr* or synov* or pericard* or amniot*)) or sample* or specimen* or biospecimen* or biosampl* or fluid* or blood or plasma or serum or urine or saliva or sputum or stool or biopsy or tissue or lavage or pus or marrow or bile or ascit* or semen or humor)) ) AND AB ( ((bio* N2 (sample* or specimen* or fluid*)) or (swab* N2 (throat or skin or cervic* or urethr* or conjuctiv*)) or (fluid* N2 (pleur* or cerebr* or synov* or pericard* or amniot*)) or sample* or specimen* or biospecimen* or biosampl* or fluid* or blood or plasma or serum or urine or saliva or sputum or stool or biopsy or tissue or lavage or pus or marrow or bile or ascit* or semen or humor)) )
S5	TI ( (shar* or public or open or cross-study or access or request or retriev* or reuse or re-use or use* or federat* or swarm or warehouse or lake or base or mesh or repositor* or registr* or platform or management) ) AND AB ( (shar* or public or open or cross-study or access or request or retriev* or reuse or re-use or use* or federat* or swarm or warehouse or lake or base or mesh or repositor* or registr* or platform or management) )
S6	TI ( ((impact* or improv* or effect* or benefit* or advance* or translation or chang* or innovate* or novel or new or emerg* or build* or gain* or progress* or promot* or strength* or cultivat* or encourage* or improv* or breakthrough* or discover* or accelerat* or intervent* or headway* or success* or facilitat* or proceed or trust)) ) AND AB ( ((impact* or improv* or effect* or benefit* or advance* or translation or chang* or innovate* or novel or new or emerg* or build* or gain* or progress* or promot* or strength* or cultivat* or encourage* or improv* or breakthrough* or discover* or accelerat* or intervent* or headway* or success* or facilitat* or proceed or trust)) )
S7	TI ( ((harm* or prevent* or stymie* or obviate or end or loss or impair* or misuse* or hurt or abuse* or injure* or block* or arrest*)) ) AND AB ( ((harm* or prevent* or stymie* or obviate or end or loss or impair* or misuse* or hurt or abuse* or injure* or block* or arrest*)) )
S8	TI ( ((economic* or (cost* N1 financ*) or reimburs* or (health N1 polic*) or (resource N1 allocat*) or expenditure*)) ) AND AB ( ((economic* or (cost* N1 financ*) or reimburs* or (health N1 polic*) or (resource N1 allocat*) or expenditure*)) )
S9	(S1 OR S2 OR S3 OR S4) AND S5
S10	S6 OR S7 OR S8
S11	S9 AND S10 (Limiters – Human)

**Table 1c.** Web of Science (Core Collection) search strategy.

1	TI=((((health or patient or participant or study or cohort or biobank* or biorepositor* or biospecimen* or biosampl* or medical or trial* or RCT* or intervention* or clinical or gen* or metabo* or proteo* or immuno* or transcripto* or imag* or electronic) NEAR/3 (data or research or sequence* or information or metadata or record*))) NEAR/3 ((shar* or public or open or cross-study or access or request or retriev* or reuse or re-use or use* or federat* or swarm or warehouse or lake or base or mesh or repositor* or registr* or platform or management)) NEAR/3 (((impact* or improv* or effect* or benefit* or advance* or translation or chang* or innovate* or novel or new or emerg* or build* or gain* or progress* or promot* or strength* or cultivat* or encourage* or improv* or breakthrough* or discover* or accelerat* or intervent* or headway* or success* or facilitat* or proceed or trust))))
2	AB=((((health or patient or participant or study or cohort or biobank* or biorepositor* or biospecimen* or biosampl* or medical or trial* or RCT* or intervention* or clinical or gen* or metabo* or proteo* or immuno* or transcripto* or imag* or electronic) NEAR/3 (data or research or sequence* or information or metadata or record*))) NEAR/3 ((shar* or public or open or cross-study or access or request or retriev* or reuse or re-use or use* or federat* or swarm or warehouse or lake or base or mesh or repositor* or registr* or platform or management)) NEAR/3 (((impact* or improv* or effect* or benefit* or advance* or translation or chang* or innovate* or novel or new or emerg* or build* or gain* or progress* or promot* or strength* or cultivat* or encourage* or improv* or breakthrough* or discover* or accelerat* or intervent* or headway* or success* or facilitat* or proceed or trust))))
3	#1 AND #2
4	TI=((((health or patient or participant or study or cohort or biobank* or biorepositor* or biospecimen* or biosampl* or medical or trial* or RCT* or intervention* or clinical or gen* or metabo* or proteo* or immuno* or transcripto* or imag* or electronic) NEAR/3 (data or research or sequence* or information or metadata or record*))) NEAR/3 ((shar* or public or open or cross-study or access or request or retriev* or reuse or re-use or use* or federat* or swarm or warehouse or lake or base or mesh or repositor* or registr* or platform or management)) NEAR/3 (((harm* or prevent* or stymie* or obviate or end or loss or impair* or misuse* or hurt or abuse* or injure* or block* or arrest*))))
5	AB=((((health or patient or participant or study or cohort or biobank* or biorepositor* or biospecimen* or biosampl* or medical or trial* or RCT* or intervention* or clinical or gen* or metabo* or proteo* or immuno* or transcripto* or imag* or electronic) NEAR/3 (data or research or sequence* or information or metadata or record*))) NEAR/3 ((shar* or public or open or cross-study or access or request or retriev* or reuse or re-use or use* or federat* or swarm or warehouse or lake or base or mesh or repositor* or registr* or platform or management)) NEAR/3 (((harm* or prevent* or stymie* or obviate or end or loss or impair* or misuse* or hurt or abuse* or injure* or block* or arrest*))))
6	#4 AND #5
7	TI=((((health or patient or participant or study or cohort or biobank* or biorepositor* or biospecimen* or biosampl* or medical or trial* or RCT* or intervention* or clinical or gen* or metabo* or proteo* or immuno* or transcripto* or imag* or electronic) NEAR/3 (data or research or sequence* or information or metadata or record*))) NEAR/3 ((shar* or public or open or cross-study or access or request or retriev* or reuse or re-use or use* or federat* or swarm or warehouse or lake or base or mesh or repositor* or registr* or platform or management)) NEAR/3 (economic* OR (cost* NEAR/1 financ*) OR reimburs* OR (health NEAR/3 polic*) OR (resource NEAR/1 allocat*) OR expenditure*))
8	AB=((((health or patient or participant or study or cohort or biobank* or biorepositor* or biospecimen* or biosampl* or medical or trial* or RCT* or intervention* or clinical or gen* or metabo* or proteo* or immuno* or transcripto* or imag* or electronic) NEAR/3 (data or research or sequence* or information or metadata or record*))) NEAR/3 ((shar* or public or open or cross-study or access or request or retriev* or reuse or re-use or use* or federat* or swarm or warehouse or lake or base or mesh or repositor* or registr* or platform or management)) NEAR/3 (economic* OR (cost* NEAR/1 financ*) OR reimburs* OR (health NEAR/3 polic*) OR (resource NEAR/1 allocat*) OR expenditure*))
9	#7 AND #8
10	TI=((((bio* NEAR/2 (medical or sample* or specimen* or repositor* or bank*)) NEAR/3 (data or research or sequence* or information or metadata or record*))) NEAR/3 ((shar* or public or open or cross-study or access or request or retriev* or reuse or re-use or use* or federat* or swarm or warehouse or lake or base or mesh or repositor* or registr* or platform or management)) NEAR/3 (((impact* or improv* or effect* or benefit* or advance* or translation or chang* or innovate* or novel or new or emerg* or build* or gain* or progress* or promot* or strength* or cultivat* or encourage* or improv* or breakthrough* or discover* or accelerat* or intervent* or headway* or success* or facilitat* or proceed or trust))))
11	AB=((((bio* NEAR/2 (medical or sample* or specimen* or repositor* or bank*)) NEAR/3 (data or research or sequence* or information or metadata or record*))) NEAR/3 ((shar* or public or open or cross-study or access or request or retriev* or reuse or re-use or use* or federat* or swarm or warehouse or lake or base or mesh or repositor* or registr* or platform or management)) NEAR/3 (((impact* or improv* or effect* or benefit* or advance* or translation or chang* or innovate* or novel or new or emerg* or build* or gain* or progress* or promot* or strength* or cultivat* or encourage* or improv* or breakthrough* or discover* or accelerat* or intervent* or headway* or success* or facilitat* or proceed or trust))))
12	#10 OR #11
13	TI=((((bio* NEAR/2 (medical or sample* or specimen* or repositor* or bank*)) NEAR/3 (data or research or sequence* or information or metadata or record*))) NEAR/3 ((shar* or public or open or cross-study or access or request or retriev* or reuse or re-use or use* or federat* or swarm or warehouse or lake or base or mesh or repositor* or registr* or platform or management)) NEAR/3 (((harm* or prevent* or stymie* or obviate or end or loss or impair* or misuse* or hurt or abuse* or injure* or block* or arrest*))))
14	AB=((((bio* NEAR/2 (medical or sample* or specimen* or repositor* or bank*)) NEAR/3 (data or research or sequence* or information or metadata or record*))) NEAR/3 ((shar* or public or open or cross-study or access or request or retriev* or reuse or re-use or use* or federat* or swarm or warehouse or lake or base or mesh or repositor* or registr* or platform or management)) NEAR/3 (((harm* or prevent* or stymie* or obviate or end or loss or impair* or misuse* or hurt or abuse* or injure* or block* or arrest*))))
15	#13 OR #14
16	TI=((((bio* NEAR/2 (medical or sample* or specimen* or repositor* or bank*)) NEAR/3 (data or research or sequence* or information or metadata or record*))) NEAR/3 ((shar* or public or open or cross-study or access or request or retriev* or reuse or re-use or use* or federat* or swarm or warehouse or lake or base or mesh or repositor* or registr* or platform or management)) NEAR/3 (economic* OR (cost* NEAR/1 financ*) OR reimburs* OR (health NEAR/3 polic*) OR (resource NEAR/1 allocat*) OR expenditure*))
17	AB=((((bio* NEAR/2 (medical or sample* or specimen* or repositor* or bank*)) NEAR/3 (data or research or sequence* or information or metadata or record*))) NEAR/3 ((shar* or public or open or cross-study or access or request or retriev* or reuse or re-use or use* or federat* or swarm or warehouse or lake or base or mesh or repositor* or registr* or platform or management)) NEAR/3 (economic* OR (cost* NEAR/1 financ*) OR reimburs* OR (health NEAR/3 polic*) OR (resource NEAR/1 allocat*) OR expenditure*))
18	#16 OR #17
19	TI=(((*omic)) NEAR/3 ((shar* or public or open or cross-study or access or request or retriev* or reuse or re-use or use* or federat* or swarm or warehouse or lake or base or mesh or repositor* or registr* or platform or management)) NEAR/3 (((impact* or improv* or effect* or benefit* or advance* or translation or chang* or innovate* or novel or new or emerg* or build* or gain* or progress* or promot* or strength* or cultivat* or encourage* or improv* or breakthrough* or discover* or accelerat* or intervent* or headway* or success* or facilitat* or proceed or trust))))
20	AB=(((omic)) NEAR/3 ((shar* or public or open or cross-study or access or request or retriev* or reuse or re-use or use* or federat* or swarm or warehouse or lake or base or mesh or repositor* or registr* or platform or management)) NEAR/3 (((impact* or improv* or effect* or benefit* or advance* or translation or chang* or innovate* or novel or new or emerg* or build* or gain* or progress* or promot* or strength* or cultivat* or encourage* or improv* or breakthrough* or discover* or accelerat* or intervent* or headway* or success* or facilitat* or proceed or trust))))
21	#19 AND #20
22	TI=(((*omic)) NEAR/3 ((shar* or public or open or cross-study or access or request or retriev* or reuse or re-use or use* or federat* or swarm or warehouse or lake or base or mesh or repositor* or registr* or platform or management)) NEAR/3 (((harm* or prevent* or stymie* or obviate or end or loss or impair* or misuse* or hurt or abuse* or injure* or block* or arrest*))))
23	AB=(((omic)) NEAR/3 ((shar* or public or open or cross-study or access or request or retriev* or reuse or re-use or use* or federat* or swarm or warehouse or lake or base or mesh or repositor* or registr* or platform or management)) NEAR/3 (((harm* or prevent* or stymie* or obviate or end or loss or impair* or misuse* or hurt or abuse* or injure* or block* or arrest*))))
24	#22 AND #23
25	TI=(((*omic)) NEAR/3 ((shar* or public or open or cross-study or access or request or retriev* or reuse or re-use or use* or federat* or swarm or warehouse or lake or base or mesh or repositor* or registr* or platform or management)) NEAR/3 (economic* OR (cost* NEAR/1 financ*) OR reimburs* OR (health NEAR/3 polic*) OR (resource NEAR/1 allocat*) OR expenditure*))
26	AB=(((omics)) NEAR/3 ((shar* or public or open or cross-study or access or request or retriev* or reuse or re-use or use* or federat* or swarm or warehouse or lake or base or mesh or repositor* or registr* or platform or management)) NEAR/3 (economic* OR (cost* NEAR/1 financ*) OR reimburs* OR (health NEAR/3 polic*) OR (resource NEAR/1 allocat*) OR expenditure*))
27	#25 AND #26
28	TI= (((bio* NEAR/2 (sample* or specimen* or fluid*)) or (swab* NEAR/2 (throat or skin or cervic* or urethr* or conjuctiv*)) or (fluid* NEAR/2 (pleur* or cerebr* or synov* or pericard* or amniot*)) or sample* or specimen* or biospecimen* or biosampl* or fluid* or blood or plasma or serum or urine or saliva or sputum or stool or biopsy or tissue or lavage or pus or marrow or bile or ascit* or semen or humor) NEAR/3 (shar* or public or open or cross-study or access or request or retriev* or reuse or re-use or use* or federat* or swarm or warehouse or lake or base or mesh or repositor* or registr* or platform or management) NEAR/3 (impact* or improv* or effect* or benefit* or advance* or translation or chang* or innovate* or novel or new or emerg* or build* or gain* or progress* or promot* or strength* or cultivat* or encourage* or improv* or breakthrough* or discover* or accelerat* or intervent* or headway* or success* or facilitat* or proceed or trust))
29	AB= (((bio* NEAR/2 (sample* or specimen* or fluid*)) or (swab* NEAR/2 (throat or skin or cervic* or urethr* or conjuctiv*)) or (fluid* NEAR/2 (pleur* or cerebr* or synov* or pericard* or amniot*)) or sample* or specimen* or biospecimen* or biosampl* or fluid* or blood or plasma or serum or urine or saliva or sputum or stool or biopsy or tissue or lavage or pus or marrow or bile or ascit* or semen or humor) NEAR/3 (shar* or public or open or cross-study or access or request or retriev* or reuse or re-use or use* or federat* or swarm or warehouse or lake or base or mesh or repositor* or registr* or platform or management) NEAR/3 (impact* or improv* or effect* or benefit* or advance* or translation or chang* or innovate* or novel or new or emerg* or build* or gain* or progress* or promot* or strength* or cultivat* or encourage* or improv* or breakthrough* or discover* or accelerat* or intervent* or headway* or success* or facilitat* or proceed or trust))
30	#28 AND #29
31	TI= (((bio* NEAR/2 (sample* or specimen* or fluid*)) or (swab* NEAR/2 (throat or skin or cervic* or urethr* or conjuctiv*)) or (fluid* NEAR/2 (pleur* or cerebr* or synov* or pericard* or amniot*)) or sample* or specimen* or biospecimen* or biosampl* or fluid* or blood or plasma or serum or urine or saliva or sputum or stool or biopsy or tissue or lavage or pus or marrow or bile or ascit* or semen or humor) NEAR/3 (shar* or public or open or cross-study or access or request or retriev* or reuse or re-use or use* or federat* or swarm or warehouse or lake or base or mesh or repositor* or registr* or platform or management) NEAR/3 (harm* or prevent* or stymie* or obviate or end or loss or impair* or misuse* or hurt or abuse* or injure* or block* or arrest*))
32	AB= (((bio* NEAR/2 (sample* or specimen* or fluid*)) or (swab* NEAR/2 (throat or skin or cervic* or urethr* or conjuctiv*)) or (fluid* NEAR/2 (pleur* or cerebr* or synov* or pericard* or amniot*)) or sample* or specimen* or biospecimen* or biosampl* or fluid* or blood or plasma or serum or urine or saliva or sputum or stool or biopsy or tissue or lavage or pus or marrow or bile or ascit* or semen or humor) NEAR/3 (shar* or public or open or cross-study or access or request or retriev* or reuse or re-use or use* or federat* or swarm or warehouse or lake or base or mesh or repositor* or registr* or platform or management) NEAR/3 (harm* or prevent* or stymie* or obviate or end or loss or impair* or misuse* or hurt or abuse* or injure* or block* or arrest*))
33	#31 AND #32
34	TI= (((bio* NEAR/2 (sample* or specimen* or fluid*)) or (swab* NEAR/2 (throat or skin or cervic* or urethr* or conjuctiv*)) or (fluid* NEAR/2 (pleur* or cerebr* or synov* or pericard* or amniot*)) or sample* or specimen* or biospecimen* or biosampl* or fluid* or blood or plasma or serum or urine or saliva or sputum or stool or biopsy or tissue or lavage or pus or marrow or bile or ascit* or semen or humor) NEAR/3 (shar* or public or open or cross-study or access or request or retriev* or reuse or re-use or use* or federat* or swarm or warehouse or lake or base or mesh or repositor* or registr* or platform or management) NEAR/3 (economic* OR (cost* NEAR/1 financ*) OR reimburs* OR (health NEAR/3 polic*) OR (resource NEAR/1 allocat*) OR expenditure*))
35	AB= (((bio* NEAR/2 (sample* or specimen* or fluid*)) or (swab* NEAR/2 (throat or skin or cervic* or urethr* or conjuctiv*)) or (fluid* NEAR/2 (pleur* or cerebr* or synov* or pericard* or amniot*)) or sample* or specimen* or biospecimen* or biosampl* or fluid* or blood or plasma or serum or urine or saliva or sputum or stool or biopsy or tissue or lavage or pus or marrow or bile or ascit* or semen or humor) NEAR/3 (shar* or public or open or cross-study or access or request or retriev* or reuse or re-use or use* or federat* or swarm or warehouse or lake or base or mesh or repositor* or registr* or platform or management) NEAR/3 (economic* OR (cost* NEAR/1 financ*) OR reimburs* OR (health NEAR/3 polic*) OR (resource NEAR/1 allocat*) OR expenditure*))
36	#34 AND #35
37	#3 OR #6 OR #9 OR #12 OR #15 OR #18 OR #21 OR #24 OR #27 OR #30 OR #33 OR #36

### Stage 3: Study selection and eligibility criteria

Two reviewers will conduct the title and abstract screening and full-text review independently. We will update the search strategy if needed to explore emerging questions identified during the title/abstract screening or full-text review per the iterative nature of a scoping review. Title-abstract screening, full-text screening, and data extraction will be managed in Covidence (RRID:SCR_016484) ^19^. Inter-reviewer disagreements in the title-abstract or full-text screening will be resolved by consensus.

***Inclusion criteria***

Included studies will be research, reports, or white papers that describe the measurement of or approaches to the measurement of the cost, harms, or benefits of sharing or otherwise reusing, as through federated learning, biomedical research data which can include clinical-epidemiological data, genetic data, high-dimensional imaging data, and human-derived biological samples and related metadata. We will not limit studies by date, geography, or language of publication.

***Exclusion criteria***

 Editorials or commentaries will be excluded. Studies or reports that focus only on the sharing pathogen data or pathogen samples, without a linkage to human data will be excluded. We will not include individual metrics related to data sharing that are not tied to a publication or report (e.g., number of datasets downloaded as reported on a data reuse platform).

### Stage 4: Charting the data

We will develop and pilot a data extraction form with three included articles to facilitate the descriptive synthesis of scoping review findings. Table 2 presents preliminary data extraction fields that will be updated during the scoping review. We will not conduct a formal assessment of the quality of included studies. The assessment of study quality varies by study type and objective. Given the wide scope of this review and the current lack of understanding of the types of studies that will be included, how we would assess the quality of included studies cannot be determined at this time and is not an important objective of this scoping review. That said, we will assess the degree of selection of the data to support the study findings, e.g., are these highly selected case studies, or do researchers consider the potential harms and benefits, is evidence for benefits, harms, and cost based on self-report or verifiable metrics like program budgets. This qualitative exploration of sources of bias will provide some information on the quality of reporting in reviewed studies and reports.

**Table 2.** Data charting fields.

**Study information**	• Author • Publication year • Study design (e.g., case study, observational, intervention; qualitative, quantitative, mixed methods; type of economic analyses) • Main objective or research question • Funder (private, public, funder name and location)
**Population (for primary** **research studies)**	• Location • Year of study, year of publication • Participant, data, or sample inclusion or exclusion criteria • Source population for data or samples • Selection process
**Measurement of or sample data sharing**	• What data are shared • Quantity of data (N, other markers of size) • Markers of dataset value • FAIRness of dataset • Markers of data quality • When data are shared • In real-time or close to real-time • After finishing data collection • After the publication of related papers With whom data are shared • How data are shared • Sent between investigators • Uploaded to a data lake or data verse (minimal effort) • Uploaded to registry or platform that requires harmonization (significant effort) • Trusted research environment (can be harmonized or not) How data on data or sample reuse are collected (measurement method) • Subjective measures ○ Personal communication ○ A longitudinal or cross-sectional survey ○ Data sharing statements Objective measures ○ Datasets uploaded ○ Datasets downloaded ○ Citations of dataset
**Measurement of costs of data or sample** **sharing**	• Short and long-term cost or cost savings (personnel costs, equipment costs, participant costs, logistical costs, including sample storage and transport, costs for standardizing or anonymizing data or for metadata creation, sample processing costs, data storage costs or costs for data access management, data reuse platform or registry costs, costs associated with regulatory compliance and agreements or licensing feeds, ethics review, or privacy-related safeguards, costs in training or otherwise supporting end users of the data or samples, cost savings related to time saved by reuse or by avoiding duplication of efforts) • Cost of harm to individuals, researchers, universities, health authorities or cost saving related to benefits to individuals, research groups, universities, health authorities
**Measurement of benefits of** **data or sample sharing**	What are the positive effects of data sharing on: • Researchers’ careers and institutions • Funding (new funders in new domains or geographies, number of grants, the amount received) • Publication rate • Impact factor of journals manuscripts published in • Research breadth and depth • Prevent spurious research & associated savings in funding/research community burnout • Lead to new fields of inquiry • Individual research participants or patients or their communities • Improving clinical care or diagnosis of rare diseases • Improved access to diagnostics, prophylactics, treatments
**Measurement of harms of data** **sharing**	What are the adverse effects of data sharing on: • Researchers’ careers, institutions • Funding (number of grants, amount received) • Publication rate • Impact factor of journals manuscripts published in • Research breadth and depth • Spurious research & associated costs to funding/research community burnout • Cost to researchers of implementing FAIR, governance development, repository deposition • Individual research participants or patients or their communities: • Reidentification of individuals and groups • Harms associated with reidentification (e.g., denial of insurance coverage, increased insurance premiums)
**Sources of bias**	• Selection of study population (e.g., how are case studies selected) • Self-report versus verifiable metrics • Reporting method
**Stakeholder concerns**	• Summary of concerns related to benefits, harms, or cost of biomedical data and sample reuse included in publications or reports from diverse stakeholders, including: ERCs, funders, DPOs, patient advocacy groups, MoHs, cross-national health authorities (e.g., WHO, US CDC, E-CDC), patient groups, indigenous or other marginalized communities, etc.
**Evidence gaps**	• Summarize areas for future research

E-CDC, European Centre for Disease Prevention and Control; ERCs, ethics review committees; DPOs, data protection officers; FAIR, findable, accessible, interoperable, and reusable; MoHs, ministries of health; US CDC, United States Centers for Disease Control and Prevention; WHO, World Health Organization.

### Stage 5: Collating, summarising, and reporting results

We will report on the screening and inclusion process using a PRISMA diagram. The data charting work will be summarized in tables and narrative form. The results summary will present both quantitative and qualitative aspects of included studies.

### Stage 6: Consultation, patient & public involvement

We will present the summary of findings from this review to a panel of stakeholders, including research funders, policymakers, and journals, to develop priority areas for improving data-sharing metrics. Specifically, we will present findings to stakeholders engaged in the RDA, CERCLE, and the GloPID-R Data Sharing Group. We will share research findings with the general public through a webinar on the Global Health Network (TGHN) platform, and through the websites of partner institutions, including the European Clinical Research Alliance on Infectious Diseases (ECRAID) and the Heidelberg Institute for Global Health (HIGH).

## Study status

We present the scoping review status and timeline in Table 3.

**Table 3.** Scoping review status at time of protocol registration and anticipated timeline.

Step	Anticipated completion date
Preliminary search	Completed October 2024
Pilot of search strategy	Completed October 2024
Implementation of search	December 2024
Title-abstract screening	April 2025
Full text screening	July 2025
Development and piloting of data charting tool	August 2025
Charting the data	November 2025
Publication of results	February 2026

## Conclusion

In this scoping review, we will identify and characterize measures of data sharing and its related costs, benefits, and harms to research participants and their source communities, researchers and their institutions, health authorities, health research funders, the Open Science community, and the general public. Costs of data sharing could include lost opportunities for publication, identification of research participants, and misinterpretation of the data. The benefits of data sharing may consist of ensuring the reliability and reproducibility of studies, identifying gaps in the research landscape, and improving the evidence base used to make regulatory, funding, and clinical decisions.

If we cannot measure and describe the costs, benefits, and harms of data reuse, we as researchers cannot inform patients and participants about the risks and benefits of data reuse, funders cannot identify and support successful data reuse efforts, and regulatory authorities, including ERCs and DPOs, cannot identify and address harms. This scoping review will summarize the existing state of research as a much-needed first step towards developing actionable, verifiable metrics for determining data sharing costs, benefits, and harms to maximize the return and minimize the harms of data sharing across stakeholder groups in the data reuse and research translation landscape.

## Ethics and dissemination

This scoping review summarizes findings from existing publications in peer-reviewed journals and does not require ethics review. Results will be disseminated through an open-access publication.

## Reporting guidelines

This scoping review protocol was developed in keeping with the PRISMA-P guidelines ^16^. This scoping review will report findings per the PRISMA-ScR guidelines ^17^.

## Data Availability

No data are associated with this article. Open Science Foundation: PRISMA-P checklist for “How do we measure the costs, benefits, and harms of sharing data from biomedical studies? A scoping review.” for https://osf.io/3dngr.
