# Supplementary material for: How do we measure the costs, benefits, and harms of sharing data from biomedical studies? A protocol for a scoping review
Source: Open Res Eur. 2025 Jan 14;3:151. Originally published 2023 Sep 18. [Version 2] doi: 10.12688/openreseurope.16063.2 (PMC11757919; doi:10.12688/openreseurope.16063.2)
Supplement: Peer review history

# Reviewer report: Reviewer response for version 2

**DOI:** 10.21956/openreseurope.20881.r50092

**Published:** 2025-02-17

**Authors:** Santiago Timón-Reina

**Affiliations:** 1 Department of Neurology, Akershus University Hospital, Lørenskog, Akershus, Norway

The authors present a protocol for a scoping review that aims to assess both qualitative and quantitative aspects of biomedical data sharing by examining its positive impacts, potential harms, and associated data and sample reuse costs.

 The methodology is designed in accordance with the PRISMA-P and PRISMA-ScR guidelines, and it specifies a predefined set of target questions covering metrics and evidence for benefits, harms, and costs.

 The protocol details the selection of key scientific databases, provides clear search terms, and outlines the anticipated outcomes of the review.

 Although I was invited to contribute at a relatively late stage in the review process, I find that the protocol is scientifically sound and thoroughly addresses the evaluation of biomedical data-sharing initiatives.

 I think this research question is both timely and significant, particularly in light of recent technological advancements and the challenges associated with data-sharing initiatives in the biomedical field.

Is the study design appropriate for the research question?

Yes

Is the rationale for, and objectives of, the study clearly described?

Yes

Are sufficient details of the methods provided to allow replication by others?

Yes

Are the datasets clearly presented in a useable and accessible format?

Not applicable

Reviewer Expertise:

Artificial Intelligence, Ontologies, Knowledge Graphs, Biomedical Data Management

I confirm that I have read this submission and believe that I have an appropriate level of expertise to confirm that it is of an acceptable scientific standard.

# Reviewer report: Reviewer response for version 2

**DOI:** 10.21956/openreseurope.20881.r50096

**Published:** 2025-02-05

**Authors:** Stefano Canali

**Affiliations:** 1 Politecnico di Milano, Milano, MI, Italy

I think the plan for this study is a great shape and indeed we need more research on this topic. I cannot really comment much on the structure and methodology of the review, but I would suggest something that I think is worth highlighting as a focus and limitation of the study. 

 The study aims at reviewing papers that *measure* costs, benefits, harms of sharing biomedical data. As I said this is a very good plan, but I think it's important to consider that many publications will be left out of this – most significantly, publications that present and discuss but do not measure costs, benefits, harms. There is a very abundant literature on this and it's clearly relevant for the topic that the authors intend to consider. 

 Just one quick example here: this paper on what the authors call burdens of data from sharing wearable data (refer 1) is clearly relevant for the topic, but does not measure these costs, benefits, harms and thus I guess as a result of the focus of the study it will be left out? Why, if so? Is this a problem or a limitation of the study?

 I think that the authors could consider their focus on measurement and justify it more clearly – I can see why the authors want to focus on measurement, but I wonder if there might be a way to involve also more qualitative papers or why they should be left out.

Is the study design appropriate for the research question?

Yes

Is the rationale for, and objectives of, the study clearly described?

Partly

Are sufficient details of the methods provided to allow replication by others?

Yes

Are the datasets clearly presented in a useable and accessible format?

Yes

Reviewer Expertise:

Philosophy and ethics of science and medicine

I confirm that I have read this submission and believe that I have an appropriate level of expertise to confirm that it is of an acceptable scientific standard, however I have significant reservations, as outlined above.

## References

[1] : Interpretation as luxury: Heart patients living with data doubt, hope, and anxiety. *Big Data & Society* .2020;7(1) : 10.1177/2053951720924436 10.1177/2053951720924436

# Reviewer report: Reviewer response for version 2

**DOI:** 10.21956/openreseurope.20881.r49738

**Published:** 2025-01-23

**Authors:** Rita Banzi

**Affiliations:** 1 Center for Health Regulatory Policies, Mario Negri Institute for Pharmacological Research, Milan, Italy

I read this new version and I feel the revisions by the authors are appropriate. In my opinion the protocol can be approved.

Is the study design appropriate for the research question?

Yes

Is the rationale for, and objectives of, the study clearly described?

Partly

Are sufficient details of the methods provided to allow replication by others?

Partly

Are the datasets clearly presented in a useable and accessible format?

Not applicable

Reviewer Expertise:

Clinical research methodology, evidence synthesis, research transparency, regulation of medicines and other health interventions

I confirm that I have read this submission and believe that I have an appropriate level of expertise to confirm that it is of an acceptable scientific standard.

# Reviewer report: Reviewer response for version 2

**DOI:** 10.21956/openreseurope.20881.r49739

**Published:** 2025-01-21

**Authors:** Florian Naudet

**Affiliations:** 1 Institut Universitaire de France, Paris, France; 2 Universite de Rennes, Rennes, Brittany, France

I consider that the authors have addressed the concerns I raised in my first peer review report appropriately.

Is the study design appropriate for the research question?

Yes

Is the rationale for, and objectives of, the study clearly described?

Yes

Are sufficient details of the methods provided to allow replication by others?

Partly

Are the datasets clearly presented in a useable and accessible format?

Not applicable

Reviewer Expertise:

Clinical trials / data sharing / reproducibility / Open Science

I confirm that I have read this submission and believe that I have an appropriate level of expertise to confirm that it is of an acceptable scientific standard.

# Reviewer report: Reviewer response for version 1

**DOI:** 10.21956/openreseurope.17343.r35885

**Published:** 2023-11-01

**Authors:** Florian Naudet

**Affiliations:** 1 Institut Universitaire de France, Paris, France; 2 Universite de Rennes, Rennes, Brittany, France

I was invited to review this protocol on the 27th of oct. 2023. I think that it is quite late given the anticipated scoping review status describe on Table 3. I wonder how can my peer review can help the authors since they are expected to be charting the data at the present time. This is a very general question for both the authors but rather for the editor. I elaborate more on this point at the end of my report.

 I have the following comments. 

- Introduction : in order to have any overlap with existing reviews, did the authors perform systematic searches to explore the existence of overlapping reviews on the same topic. e.g. they cite ours (reference 6, [1]) but since then, some other might have been published. I mean that identifying all similar studies on the same topic may be helpful to develop the protocol. And perhaps one rather need an update than a new scoping review. As far as I understand this scoping review is broader than ours and I think it is better than a simple update to answer the authors question. But at least some discussion of existing reviews is needed.
- As far as the scope of this review is broader and encompass "biomedical studies" [Title], I struggled to understand why : 
  - Biomedical research conducted with data from electronic health records (EHRs) won't be selected. Perhaps these studies can be done at low cost (although this claim is not accompanied by a given reference) but these surely involve even more risk regarding sharing of their data than in comparison with clinicaltrials as such datasets may contain even more identifiable information. In addition, the benefit of sharing this data (that can be of lower quality) may also be different. I do think that it is an important aspect to consider when exploring the benefit risk of data sharing for "biomedical studies" ; 
  - Similarly, I struggled to understand why sharing high dimensional genetic data is not considered in this review. One would expect that the benefit of sharing this kind of data is better documented. One can also imagine specific risks/harms associated with these data. Reviewing this information may be of interest.
  - In fact, the title directly refers to "biomedical studies". It is not reflected by those 2 methodological choices that clearly limit the scope and do not reflect all biomedical studies. I therefore suggest to include all studies without those two restrictions. If the authors don't agree with this suggestion, the title -and the abstract- must be changed to reflect the fact that the scope is not so broad. It is quite misleading in its current form.

- It is stated that "the initial review questions were developed through consultation with the research team and feedback from researchers and stakeholders, including funding groups, who work to facilitate data reuse". This statement is not reproducible. Authors must detail : all stakeholders that were involved with details on their specific involvement (how, when they were consulted and how much change they brought in the present manuscript).   
- This is not clear to me : "Measurement ... 2. Is there a lot of difference between question 1 and 2?" What are question 1 and question 2 here? 
- In the part "Empirical Evidence", how the fears, and benefits were pre-specified? Was it based on previous literature, was it based on the insights from the various stakeholders (see my previous question)? Is it possible to imagine other shortcomings that were not pre-specified and that will emerge during the scoping review process. How those thematics will be (or were) handled during the research process? 
- I have difficulties to understand the part "New insights". Does it mean new insights in contrast with the previous literature? If yes, it looks like this new scoping review is rather and update of the previous ones and I would expect some sort of detailed presentation of the existing literature / results observed in previous scoping reviews on the topic. This comment is in line with my previous comment on the introduction. 
- Stage 2 is insufficiently described : 
  - Literature searches should be detailed for all electronic database and not solely for one (Ovid Medline). This is a protocol that must provide sufficient details to reproduce the searches ; 
  - It should follow the PRESS guideline statement [2] and involves peer review by an information specialist ; 
  - As authors state they will also explore case report from funders, research institutions etc. "We will examine grey literature ( *e.g*., case reports from funders, research, or public health institutions)." It is paramount to detail this but it is insufficiently detailed in the current version. One would expect that a list of all those institution should be provided. In my experience, data sharing platforms also provide a lot of very important metrics (YODA, Vivli, etc.). Authors should mention all initiative that will be explored. Some ideas can be found in [1].
  - When it comes to exploring funders it is paramount to make a distinction between public and private (e.g. pharmaceutical companies) funders as those have very distinct policies. Author should detail how they will approach both public and private funders. 
- I agree that study quality is not assessed in most scoping reviews. But I have the feeling that in some instance it could be useful. At least, authors should explain the reason why they find it not useful to address study quality in their scoping review ; 
- Regarding Table 2 / I would reconsider using journal impact factor as a measure of benefit or harm of data sharing. JIF has no value at the article level and there is a large body of literature about that (e.g. 3). 

I hope these comments will be helpful as this project is of importance to the field. I hope that the protocol will be changed to incorporate these changes. In this case, it needs to be very transparent regarding : the current stage of the searches at the time of the revisions. 

 In case that this feedback was too late for authors to implement the changes because their searches were already performed/ongoing, authors must i) indicate in their protocol the limitations that I have raised and ii) explicitly state in the protocol that they will write in the final paper that although the protocol was peer reviewed some of the changes were not included because the searches were already ongoing at the stage of the protocol review. Then the final paper will also have to i) explicitly state that protocol peer review does not means that all choices were endorsed because of time constraint and ii) reflect those limitations explicitly.

 In another hand if authors are able to implement all my comments, authors may consider tracking in a specific section of their final papers all changes to the protocol including those occurring during the peer review process and the reasons for the change. I think that the protocol should also mention specifically how changes to the initial protocol will be reported in the final manuscript. 

 Last, I miss a registration of the protcol (e.g. on the OSF) before the start of the searches. I was able to find the PRISMA checklist (and this is excellent) but not a pre registration. If it was not pre-registered please make it explicit. Else, please provide the number of preregistration. This is of importance since this protocol cannot be fully considered as a pre-registration since searches are ongoing at the time I am appraising it.

 I hope that these comments were useful.

Is the study design appropriate for the research question?

Yes

Is the rationale for, and objectives of, the study clearly described?

Yes

Are sufficient details of the methods provided to allow replication by others?

Partly

Are the datasets clearly presented in a useable and accessible format?

Not applicable

Reviewer Expertise:

Clinical trials / data sharing / reproducibility / Open Science

I confirm that I have read this submission and believe that I have an appropriate level of expertise to confirm that it is of an acceptable scientific standard, however I have significant reservations, as outlined above.

## References

[1] : PRESS Peer Review of Electronic Search Strategies: 2015 Guideline Statement. *J Clin Epidemiol* .2016;75: 10.1016/j.jclinepi.2016.01.021 40-6 10.1016/j.jclinepi.2016.01.021 27005575

[2] : Status, use and impact of sharing individual participant data from clinical trials: a scoping review. *BMJ Open* .2021;11(8) : 10.1136/bmjopen-2021-049228 e049228 10.1136/bmjopen-2021-049228 34408052 PMC8375721

[3] : Deep impact: unintended consequences of journal rank. *Front Hum Neurosci* .2013;7: 10.3389/fnhum.2013.00291 291 10.3389/fnhum.2013.00291 23805088 PMC3690355

# Response

**Published:** 2024-12-30

**Authors:** Lauren Maxwell

**Affiliations:** Universitätsklinikum Heidelberg, Germany

20 Dec 2024   Dear Dr. Naudet, Thank you for presenting us with a detailed review of our protocol submitted to Open Research Europe titled, “How do we measure the costs, benefits, and harms of sharing data from biomedical studies? A protocol for a scoping review.” We present below our responses and indicate the edits we have made according to your detailed comments which we believe have improved the protocol. Thank you for your continued interest in this work. **Table 1: Summary of edits and our responses** **Comment** **Response/edits** Page #; paragraph*  I was invited to review this protocol on the 27th of oct. 023. I think that it is quite late given the anticipated scoping review status describe on Table 3. I wonder how can my peer review can help the authors since they are expected to be charting the data at the present time. This is a very general question for both the authors but rather for the editor. I elaborate more on this point at the end of my report. We have revised our timeline (Table 3). We put the review on hold and then updated the search strategy to align with the reviewers’ feedback and the main objective of the review. Page 18; Table 3 “Scoping review status at time of protocol registration and anticipated timeline.” Introduction: In order to have any overlap with existing reviews, did the authors perform systematic searches to explore the existence of overlapping reviews on the same topic. e.g. they cite ours (reference 6, [1]) but since then, some other might have been published. I mean that identifying all similar studies on the same topic may be helpful to develop the protocol. And perhaps one rather need an update than a new scoping review. As far as I understand this scoping review is broader than ours and I think it is better than a simple update to answer the authors question. But at least some discussion of existing reviews is needed. We implemented our search in Ovid(Medline), limiting the search to articles with “systematic review” or “scoping review” in the title. We reviewed the 118 title-abstracts from that search and found 12 related reviews where we examined the full text. These reviews, while interesting, did not have important areas of overlap with the work we have envisioned here, and we did not see the need to cite these reviews in the protocol.   We also (re)checked the Cochrane database of systematic reviews and found no related Cochrane review on this topic. NA As far as the scope of this review is broader and encompass "biomedical studies" [Title], I struggled to understand why: Biomedical research conducted with data from electronic health records (EHRs) won't be selected. Perhaps these studies can be done at low cost (although this claim is not accompanied by a given reference) but these surely involve even more risk regarding sharing of their data than in comparison with clinical trials as such datasets may contain even more identifiable information. In addition, the benefit of sharing this data (that can be of lower quality) may also be different. I do think that it is an important aspect to consider when exploring the benefit risk of data sharing for "biomedical studies" We deleted this exclusion criteria per the reviewer’s feedback. Page 15; paragraph 3 Similarly, I struggled to understand why sharing high dimensional genetic data is not considered in this review. One would expect that the benefit of sharing this kind of data is better documented. One can also imagine specific risks/harms associated with these data. Reviewing this information may be of interest. We have not excluded high-dimensional genetic data sharing from the inclusion as it important to see if there are any differences in the costs, benefits or harms of data sharing between different data types. The inclusion criteria has been revised to say: “Included studies will be research or reports that describe approaches to measuring the cost, harms, or benefits of sharing or otherwise reusing, as through federated learning, biomedical research data which can include clinical-epidemiological data, genetic, and imaging data and metadata and metadata associated with human-derived biological samples.”   We have also amended the abstract and introduction to explicitly mention the different data/sample types: Abstract: “The benefits of sharing participant-level data, including clinical or epidemiological data, genomic data, high-dimensional imaging data, or human-derived samples…” Introduction: “Sharing data, including clinical or epidemiological (clin-epi) data, genetic data, high-dimensional imaging data, or human-derived samples and related metadata, from biomedical research…”   We also make it clear under the ‘Study rationale and objectives’ section what the difference between data types could be: “Within public health research, data sharing varies by data type. While sharing high dimensional genetic data is expected, enforced, and facilitated by widely adopted data and metadata standards, sharing other data types, like clinical-epidemiological data, is less common and, in some ways, more complex. As such, we might expect to see differences in the cost of sharing clin-epi rather than human or pathogen OMICs data.” Page 15; paragraph 2               Page 2 (abstract) Pages 2-3; paragraph 1 (introduction)       Page 4; paragraph 2 In fact, the title directly refers to "biomedical studies". It is not reflected by those 2 methodological choices that clearly limit the scope and do not reflect all biomedical studies. I therefore suggest including all studies without those two restrictions. If the authors don't agree with this suggestion, the title -and the abstract- must be changed to reflect the fact that the scope is not so broad. It is quite misleading in its current form. We believe that the title captures the objective of the research without being misleading. As clarified above and in the text, we will include studies that relate to the sharing or other reuse modalities for clin-epi data, genetic data, high-dimensional imaging data, or human-derived samples and related metadata, from biomedical research.  NA It is stated that "the initial review questions were developed through consultation with the research team and feedback from researchers and stakeholders, including funding groups, who work to facilitate data reuse". This statement is not reproducible. Authors must detail all stakeholders that were involved with details on their specific involvement (how, when they were consulted and how much change they brought in the present manuscript). We have amended the text related to stakeholder consultation, which now says the following: “The initial review questions were developed through consultation with the research team and feedback from researchers and stakeholders who participated in the Coalition for Equitable Research in Low Income Settings (CERCLE) in 2019, and in the 2019 Global Research Collaboration for Infectious Disease Preparedness (GloPID-R) Data Sharing Working Group membership who work to facilitate biomedical data reuse in the context of infectious disease and beyond. Following these stakeholder consultations, the review scope was unchanged.” Page 4; paragraph 5 This is not clear to me: "Measurement ... 2. Is there a lot of difference between question 1 and 2?" What are question 1 and question 2 here? This is a comment that should not have been included in the protocol submission.  We have reworked the section in response to reviewer feedback. Pages 4-5; paragraph 1 In the part "Empirical Evidence", how the fears, and benefits were pre-specified? Was it based on previous literature, was it based on the insights from the various stakeholders (see my previous question)? Is it possible to imagine other shortcomings that were not pre- specified and that will emerge during the scoping review process. How those thematics will be (or were) handled during the research process? We opted to do a scoping reviews as they, by design, allow for the extension of research questions based on findings in the review itself.  NA I have difficulties to understand the part "New insights". Does it mean new insights in contrast with the previous literature? If yes, it looks like this new scoping review is rather and update of the previous ones and I would expect some sort of detailed presentation of the existing literature / results observed in previous scoping reviews on the topic. This comment is in line with my previous comment on the introduction.  The phrase “New insights” refers to a potential benefit of data sharing or other methods of data reuse. It is written under the phrase:  What evidence do we have to support data-sharing-related benefits? This does not refer to updating previous reviews rather to whether data sharing or other forms of reuse lead to new scientific or clinical insights. To clarify this, we have amended the text under the reworked section “1.3. What evidence supports the benefits of biomedical data or sample sharing?” to the following:

- New scientific or clinical insights
- New opportunities for research and development (e.g., patents)

Page 5; paragraph 2 Stage 2 is insufficiently described: Literature searches should be detailed for all electronic database and not solely for one (Ovid Medline). This is a protocol that must provide sufficient details to reproduce the searches;  We have added the search strategies for all of the electronic databases searched  into the protocol as Tables 1a–1c.    Pages 7-15 It should follow the PRESS guideline statement [2] and involves peer review by an information specialist; All search strategies were reviewed by an information scientist at Universitätsklinikum Heidelberg Library, when the initial search was developed in 2023 and then when the revised search was developed in December 2024.   We have included the following text to detail how the search strategy was developed under the section ‘Stage 2: Identifying relevant studies’: “In keeping with the Peer Review of Electronic Search Strategies (PRESS) 2015 Guidelines, the search strategies were developed through: translating the research questions into a PICO format, using Boolean and proximity operators, applying subject headings (e.g., MeSH terms) and free-text terms, pilot testing to ensure appropriate syntax, and applying limits and filters. The search strategies were reviewed by an information scientist at Universitätsklinikum Heidelberg Library.”         Page 6; paragraph 2 As authors state they will also explore case report from funders, research institutions etc. "We will examine grey literature (e.g., case reports from funders, research, or public health institutions)." It is paramount to detail this but it is insufficiently detailed in the current version. One would expect that a list of all those institution should be provided. In my experience, data sharing platforms also provide a lot of very important metrics (YODA, Vivli, etc.). Authors should mention all initiative that will be explored. Some ideas can be found in [1]. We have updated the text under ‘Stage 2: Identifying relevant studies’ to better describe the actions we will take here.  The amended text is as follows: “We will search OpenGrey, Google, and the websites of national or international bodies whose work focuses on biomedical data sharing to identify related grey literature reports. These groups will include the Wellcome Trust, RAND corporation, Digital Research Alliance of Canada, Canadian Institutes of Health Research (CIHR), US National Institutes of Health, the European Commission, Fiocruz, Clinical Research Data Sharing Alliance (CRSDA), Research Data Alliance (RDA), World Data Systems (WDS), the Global Alliance for Genomics and Health (GA4GH), the European Bioinformatics Institute (EMBL-EBI) and the Biobanking and BioMolecular Resources Research Infrastructure (BBMRI-ERIC, the World Health Organization (WHO), CERCLE, the Infectious Diseases Data Observatory (IDDO), the European and Developing Countries Clinical Trials Partnership (EDCTP), the Global Health Network (TGHN), and the European Clinical Research Infrastructure Network (ECRIN), the Bill and Melinda Gates Foundation (BMBF), the Terry Fox Foundation, the Rare Cancer Research Foundation (RCRF), the Alzheimer’s Disease Data Initiative (ADDI), the National Organization for Rare Disorders (NORD), the Haystack Project, the Cancer Research Data Commons (CRDC), Accelerating Medicines Partnership (AMP), the Observational Health Data Sciences and Informatics (OHDSI) initiative, The Future of Research Communication and e-Scholarship (FORCE11), and Make Data Count.”   We will not search the websites of related biomedical data registries to understand what metrics these registries implement. A 6 December 2024 search of the FAIRsharing.org registry of databases indicates more the 1K databases in the biomedical space, which only considered databases that are registered on FAIRsharing.org, a small fraction of existing biomedical databases. Data reuse metrics from repositories and platforms tend to focus on the number of datasets shared and reused or the number of publications related to data reuse. In this review, our focus is on the benefits, harms, and cost of data reuse rather than on the quantity. The review and extraction of data reuse metrics from biomedical databases is beyond the scope of this work.” Page 6; paragraphs 3 and 4 When it comes to exploring funders it is paramount to make a distinction between public and private (e.g. pharmaceutical companies) funders as those have very distinct policies. Author should detail how they will approach both public and private funders. We will not explore the reports of private funders due to the potential for biased reporting. We have amended the text under ‘Stage 2: Identifying relevant studies’ to clearly specify this: “Because of the possibility of biased reporting, we will not explore the websites of pharmaceutical companies or for-profit groups for their reports on the costs, benefits, and harms of biomedical data sharing and reuse. If reports on the costs, benefits, and harms of biomedical data reuse have been funded by for-profit groups, this information will be reported in the study description (e.g. Table 1 of the scoping review publication).” Page 6; paragraph 3 and page 7; paragraph 1 I agree that study quality is not assessed in most scoping reviews. But I have the feeling that in some instance it could be useful. At least, authors should explain the reason why they find it not useful to address study quality in their scoping review    We have included the following text under ‘Stage 4: Charting the data’ to address the reviewer’s comment: “We will not conduct a formal assessment of the quality of included studies. The assessment of study quality varies by study type and objective. Given the wide scope of this review and the current lack of understanding of the types of studies that will be included, how we would assess the quality of included studies cannot be determined at this time and is not an important objective of this scoping review. That said, we will assess the degree of selection of the data to support the study findings, e.g., are these highly selected case studies, or do researchers consider the potential harms and benefits, is evidence for benefits, harms, and cost based on self-report or verifiable metrics like program budgets. This qualitative exploration of sources of bias will provide some information on the quality of reporting in reviewed studies and reports.” Page 15; paragraph 4 Regarding Table 2 / I would reconsider using journal impact factor as a measure of benefit or harm of data sharing. JIF has no value at the article level and there is a large body of literature about that (e.g. 3). We completely agree with this statement. Unfortunately, many groups, including our own university, Universitatsklinikum Heidelberg, continue to use antiquated measures of assessing researcher performance, including journal impact factor.  For example, Universitatsklinikum Heidelberg will only consider promotion for faculty who have published papers in journals with JIF in the upper one third of the JIF for their specific area in the biomedical sciences. Articles published in journals that choose not to publish their impact factor, because of how the impact factors are biased, like PLOS journals, will not be considered for academic promotion. Given that universities, like our own, continue to base their criteria for researcher advancement on JIF, we consider this as an important metric, although we agree that this is a biased and antiquated approach that is not evidence based. NA I hope these comments will be helpful as this project is of importance to the field. I hope that the protocol will be changed to incorporate these changes. In this case, it needs to be very transparent regarding: the current stage of the searches at the time of the revisions. We have updated the timeline for this scoping review (Table 3) to reflect that the searches will be initiated in December 2024 after submitting our response to reviewers. Page 18; Table 3 “Scoping review status at time of protocol registration and anticipated timeline.” In case that this feedback was too late for authors to implement the changes because their searches were already performed/ongoing, authors must i) indicate in their protocol the limitations that I have raised and ii) explicitly state in the protocol that they will write in the final paper that although the protocol was peer reviewed some of the changes were not included because the searches were already ongoing at the stage of the protocol review. Then the final paper will also have to i) explicitly state that protocol peer review does not means that all choices were endorsed because of time constraint and ii) reflect those limitations explicitly. This feedback was not too late. As stated above, we had to put this review on hold.  We have subsequently updated the search strategy and timeline (Table 3). The search will be initiated in December 2024 after submitting our response to reviewers. Page 18; Table 3 “Scoping review status at time of protocol registration and anticipated timeline.” In another hand if authors are able to implement all my comments, authors may consider tracking in a specific section of their final papers all changes to the protocol including those occurring during the peer review process and the reasons for the change. I think that the protocol should also mention specifically how changes to the initial protocol will be reported in the final manuscript. Changes were made to the protocol before initiating the reconfigured search and before beginning with title-abstract screening. These changes are reflected in the versioning in Open Science Europe. Given that the changes were made before initiating the review process, we believe that this will be sufficient. NA Last, I miss a registration of the protocol (e.g. on the OSF) before the start of the searches. I was able to find the PRISMA checklist (and this is excellent) but not a pre-registration. If it was not pre-registered, please make it explicit. Else, please provide the number of preregistrations. This is of importance since this protocol cannot be fully considered as a pre-registration since searches are ongoing at the time I am appraising it. We consider the registration on Open Research Europe the registration of the protocol.   Please note that PROSPERO will not accept the registration of protocols for scoping reviews.  Also note that the search was redone based on reviewer feedback and now the protocol on Open Research Europe represents the most recent version of the protocol. NA * Page numbers are based on the clean version.

# Reviewer report: Reviewer response for version 1

**DOI:** 10.21956/openreseurope.17343.r35186

**Published:** 2023-10-06

**Authors:** Rita Banzi

**Affiliations:** 1 Center for Health Regulatory Policies, Mario Negri Institute for Pharmacological Research, Milan, Italy

The manuscript reports on a protocol of a scoping review, focusing on qualitative or quantitative approaches for measuring the cost of data sharing or its impact. This topic is extremely relevant, considering the current interest on data sharing and the expectation of its broad application across research disciplines. From the background section it seems the authors want to focus on costs and harms, that is somehow innovative, and this work can potentially inform policies and practice on data sharing.

 In general, the protocol is well written, and its publication welcomed. However, I would suggest some revisions that the authors may will to consider to improve the clarity and transparency of the review process.

 1. Rational and objectives

 Although the general scope of the review is clear, the text is sometimes confused in stating the specific objectives of the review. The paragraph “study rational and objectives” reports a broad objective

 “to identify and describe qualitative and quantitative approaches to measuring the impact of data sharing on researchers’ careers, clinical or public health practice, and science. We will examine the types of studies and the results of those studies related to the impacts and costs of data sharing” (page 3).

 The term “impacts” may have very different meanings depending on the context, perspectives, etc. and may be misleading. Surely, it contains both positive and negative aspects so I would encourage the author to clearly define the meaning of “impact” in the context of the review. The attempt to collect evidence on the impact of data sharing may not be the same as collecting evidence on its benefit, harms, and costs.

 2. Identifying the research question

 I believe this partial misalignment is also reflected by the list of research questions (page 4).

 For instance, the first part of the questions is about metrics. Please, note that point 2 under the heading “Measurement” seems to be an error as well as point 1.i that – I assume - refers to the type of studies that the authors would like to assess to answer to this question. Moreover, point 1 and 3 partly overlap.

 The second part named “empirical evidence” aims to collect information on actual measurement of harms (or fears? Maybe this are not the same concepts, and it would be great to clarify which is the target), benefit and costs/saving.

 I would suggest revising how the questions are framed with an eye on how the results of the review will be presented in a friendly format. Considering how complex is the topic the authors are willing to explore, it would be easier to align how the questions are framed, to the data collection form and to the charting of studies/results (please see Table 2).

 For instance, the questions can be re-grouped:

 Data-sharing related benefits – metrics/empirical evidence

 Data-sharing related harms – metrics/empirical evidence

 Data sharing related costs/saving – metrics/empirical evidence

 Some more details on the identification process may increase the transparency of the protocol, e.g., who were involved in the consultations and how it was organized (survey, formal meeting, informal email exchange, etc.)

 3. Stage 2: Identifying relevant studies

 Please, state if the searches were limited by year of publication or language.

 4. Stage 3: Study selection and eligibility criteria

 Inclusion and exclusion criteria are rather vague, which is acceptable for a scoping review but maybe some details can be added. For instance, the authors can move here the information that is now in the background about the focus on clinical-epidemiological studies and state if all study designs are eligible, including case studies, surveys, metrics, and experimental studies, using qualitative or quantitative methods. I tend to disagree on the decision to exclude experience of sharing clinical routinely collected data in EHR. The benefit of sharing this type of data is going to be substantial, and also obstacles are not so negligible especially when there is the need for data base integration or to assess the consent to re-use.

 If the authors would not be able to reconsider this exclusion criteria, I would suggest providing more explanation supporting this decision.

 5. Stage 6: Consultation, patient & public involvement

 As for the identification of questions, I would encourage the authors to provide more information on this stage, how is planned, which is the scope, who will be involved, etc.

 6. Please check the numbering of the reference list, as it seems there are some mistakes (n.17- 19)

Is the study design appropriate for the research question?

Yes

Is the rationale for, and objectives of, the study clearly described?

Partly

Are sufficient details of the methods provided to allow replication by others?

Partly

Are the datasets clearly presented in a useable and accessible format?

Not applicable

Reviewer Expertise:

Clinical research methodology, evidence synthesis, research transparency, regulation of medicines and other health interventions

I confirm that I have read this submission and believe that I have an appropriate level of expertise to confirm that it is of an acceptable scientific standard, however I have significant reservations, as outlined above.

# Response

**Published:** 2024-12-30

**Authors:** Lauren Maxwell

**Affiliations:** Universitätsklinikum Heidelberg, Germany

Dear Dr. Banzi, Thank you for presenting us with a detailed review of our protocol submitted to Open Research Europe titled, “How do we measure the costs, benefits, and harms of sharing data from biomedical studies? A protocol for a scoping review.” We present below our responses and indicate the edits we have made according to your detailed comments which we believe have improved the protocol. Thank you for your continued interest in this work. **Table 1: Summary of edits and our responses** **Comment** **Response/edits** Page #; paragraph*  1. Rational and objectives   Although the general scope of the review is clear, the text is sometimes confused in stating the specific objectives of the review. The paragraph “study rational and objectives” reports a broad objective   “to identify and describe qualitative and quantitative approaches to measuring the impact of data sharing on researchers’ careers, clinical or public health practice, and science. We will examine the types of studies and the results of those studies related to the impacts and costs of data sharing” (page 3).  The term “impacts” may have very different meanings depending on the context, perspectives, etc. and may be misleading. Surely, it contains both positive and negative aspects so I would encourage the author to clearly define the meaning of “impact” in the context of the review. The attempt to collect evidence on the impact of data sharing may not be the same as collecting evidence on its benefit, harms, and costs. We have modified the text in the ‘Study rationale and objectives’ section to address the reviewers’ justified concerns here. The text now reads: “This scoping review will identify and describe qualitative and quantitative approaches to (1) measuring the positive impacts of biomedical data or sample sharing or other forms of data or sample reuse (e.g. federated data or sample reuse) on researchers’ careers, clinical or public health practice, and science; (2) the harms of data or sample sharing, including harms to investigators, research participants or patients whose routinely collected data are reused for research, or public health practice as through misuse of data for misleading inference and (3) the costs associated with data and sample reuse. We will examine the types of studies and the results of those studies related to the impacts (e.g., positive, negative, no impact) and costs of data sharing. Biomedical data sharing will be defined broadly as individual investigators sharing data on a case-by-case basis or individuals or institutions sharing data on a cross-project level through individual participant data meta-analyses (IPD-MAs), data verses, data lakes, platforms, repositories, and registries. The information collected and summarized through this scoping review will be used to develop a research agenda for measuring the harms, benefits, and costs of biomedical data and sample sharing.”   Page 3; paragraph 4 and page 4; paragraph 1         2. Identifying the research question   I believe this partial misalignment is also reflected by the list of research questions (page 4).   For instance, the first part of the questions is about metrics. Please, note that point 2 under the heading “Measurement” seems to be an error as well as point 1.i that – I assume - refers to the type of studies that the authors would like to assess to answer to this question. Moreover, point 1 and 3 partly overlap. We have taken care to ensure the text in the ‘Stage 1: Identifying the research question’ section aligns with that in the ‘Study rationale and objectives’ section. The proposed research questions now include: “ **1. Data Sharing-Related Benefits** **Metrics and Evidence**

- **1.1. What types of metrics or methods are used to measure the benefits of biomedical data or sample sharing?**
   Examples:
  - Case studies, empirical measures, modelled estimates.
- **1.2. What are the sources of data used to measure these benefits?**
   Examples:
  - Researcher or funder self-report, bibliometrics, altmetrics, dataset persistent identifiers, scientific knowledge graphs (e.g., OpenAIRE, Web of Science).

**Empirical Evidence**

- **1.3. What evidence supports the benefits of biomedical data or sample sharing?**
   Examples:
  - New scientific or clinical insights.
  - New opportunities for research and development (e.g., patents).
  - Increased funding or publication opportunities.
  - New collaborations or expanded research networks.
  - Improved study design and translation of findings into practice.
  - Prevention of unnecessary research.

**2. Data Sharing-Related Harms** **Metrics and Evidence**

- **2.1. What types of metrics or methods are used to assess the harms of biomedical data or sample sharing?**
   Examples:
  - Empirical measures, case studies, modelled estimates.

**Empirical Evidence**

- **2.2. What evidence supports concerns or fears about biomedical data or sample sharing?**
   Examples:
  - Lost opportunities for funding or publication.
  - Identification and stigmatization of participants.
  - Misinterpretation or misuse of data or samples.
  - Lack of benefit sharing with source communities.
  - Ethical concerns, especially for indigenous or marginalized populations.
  - Lack of credit or attribution for data or sample providers.

**3. Data Sharing-Related Costs and Savings** **Metrics and Evidence**

- **3.1. What types of metrics or methods are used to assess the costs and savings of biomedical data or sample sharing?**
   Examples:
  - Modelled estimates, cost-benefit analyses.
- **3.2. What are the sources of data used to evaluate costs and savings?**
   Examples:
  - Institutional financial records, funding reports, researcher self-report.

**Empirical Evidence**

- **3.3. What evidence exists about the costs or savings associated with sharing or not sharing biomedical data or samples?**
   Examples:
  - Direct and indirect costs for investigators, institutions, and funders.
  - Cost-effectiveness or savings from avoided duplication of research.”

Pages 4-6 The second part named “empirical evidence” aims to collect information on actual measurement of harms (or fears? Maybe this are not the same concepts, and it would be great to clarify which is the target), benefit and costs/saving. We separated the research questions by whether they are related to measurement or evidence. These concepts are related, but we wanted to separate them out to be able to understand how the benefits, harms, and cost of biomedical data and sample reuse are measured and then what sources of data are used to measure benefits, costs, and harms of biomedical data and sample reuse. Pages 4-6 I would suggest revising how the questions are framed with an eye on how the results of the review will be presented in a friendly format. Considering how complex is the topic the authors are willing to explore, it would be easier to align how the questions are framed, to the data collection form and to the charting of studies/results (please see Table 2). We have tried to revise how the questions are written and have taken care to ensure that Table 2 (data charting) aligns with the draft review questions. Please note that, in a scoping review, the review questions and charting fields may change as we learn more about the state and scope of the included literature. Pages 4-6 (research questions) Pages 15-17 (table 2) For instance, the questions can be re-grouped: Data-sharing related benefits – metrics/empirical evidence Data-sharing related harms – metrics/empirical evidence Data sharing related costs/saving – metrics/empirical evidence Some more details on the identification process may increase the transparency of the protocol, e.g., who were involved in the consultations and how it was organized (survey, formal meeting, informal email exchange, etc.) The questions have been reorganized into three categories—benefits, harms, and costs—to align with the scoping review's objectives and facilitate data collection and results charting. Each question now distinctly focuses on metrics or empirical evidence. We hope that these changes align with the reworking of the questions that the reviewer was referring to here.   Pages 4-6

 3. **Stage 2: Identifying relevant studies   Please, state if the searches were limited by year of publication or language.**

 We have added a statement on this to the section on ‘Identifying Relevant Studies’.   Specifically, we included the statement: “Searches will not be limited by date or language of publication.” Page 7; paragraph 2

 4. **Stage 3: Study selection and eligibility criteria   Inclusion and exclusion criteria are rather vague, which is acceptable for a scoping review but maybe some details can be added. For instance, the authors can move here the information that is now in the background about the focus on clinical-epidemiological studies and state if all study designs are eligible, including case studies, surveys, metrics, and experimental studies, using qualitative or quantitative methods.     I tend to disagree on the decision to exclude experience of sharing clinical routinely collected data in EHR. The benefit of sharing this type of data is going to be substantial, and also obstacles are not so negligible especially when there is the need for data base integration or to assess the consent to re-use. If the authors would not be able to reconsider this exclusion criteria, I would suggest providing more explanation supporting this decision.**

 We have added further detail and clarified the inclusion and exclusion criteria. We have removed the exclusion criteria related to the exclusion of studies that only report on the use of EHR data from one system.   The inclusion/exclusion criteria now read: ***Inclusion criteria*** Included studies will be research, reports, or white papers that describe the measurement of or approaches to the measurement of the cost, harms, or benefits of sharing or otherwise reusing, as through federated learning, biomedical research data which can include clinical-epidemiological data, genetic data, high-dimensional imaging data, and  human-derived biological samples and related metadata. We will not limit studies by date, geography, or language of publication. ***Exclusion criteria*** Editorials or commentaries will be excluded. Studies or reports that focus only on the sharing pathogen data or pathogen samples, without a linkage to human data will be excluded. We will not include individual metrics related to data sharing that are not tied to a publication or report (e.g., number of datasets downloaded as reported on a data reuse platform).   Page 15; paragraphs 2 and 3

**5. Stage 6: Consultation, patient & public involvement   As for the identification of questions, I would encourage the authors to provide more information on this stage, how is planned, which is the scope, who will be involved, etc.**  

 We have revised this text to include additional details: “We will present the summary of findings from this review to a panel of stakeholders, including research funders, policymakers, and journals, to develop priority areas for improving data-sharing metrics. Specifically, we will present findings to stakeholders engaged in the RDA, CERCLE, and the GloPID-R Data Sharing Group. We will share research findings with the general public through a webinar on the Global Health Network (TGHN) platform, and through the websites of partner institutions, including the European Clinical Research Alliance on Infectious Diseases (ECRAID) and the Heidelberg Institute for Global Health (HIGH).” Page 17; paragraph 2 and page 18; paragraph 1

**6. Please check the numbering of the reference list, as it seems there are some mistakes (n.17- 19)**

 We have gone through and fixed the referencing.  Pages 19-20 *Page numbers correspond to the clean version
